# Photonic integrated field-programmable disk array signal processor

**DOI:** 10.1038/s41467-019-14249-0

**Published:** 2020-01-21

**Authors:** Weifeng Zhang, Jianping Yao

**Affiliations:** Microwave Photonic Research Laboratory, School of Electrical Engineering and Computer Science, University of Ottawa, 25 Templeton Street, Ottawa, ON K1N 6N5 Canada

**Keywords:** Electrical and electronic engineering, Microwave photonics, Microresonators

## Abstract

Thanks to the nature of strong programmability, field-programmable gate arrays (FPGAs) have been playing a significant role in signal processing and control. With the explosive growth in digital data, big data analytics becomes an important emerging field, in which FPGAs are a major player. However, the computational speed and power efficiency provided by FPGAs are limited by electronic clock rates and Ohmic losses. To overcome the limitations, photonics is envisioned as an enabling solution, thanks to its ultrafast and low power consumption feature. In this paper, we propose a scalable photonic field-programmable disk array (FPDA) signal processor. Ultra-compact microdisk resonators are leveraged as a fundamental execution units in the core to route, store and process optical signals. By field-programming the processor, diverse circuit topologies can be realized to perform multiple specific signal processing functions including filtering, temporal differentiation, time delay, beamforming, and spectral shaping.

## Abstract (web summary)

FPGAs have long been of interest as reconfigurable circuits, but their electronic nature provides eventual limitations. Here the authors demonstrate a photonic digital signal processor that is field programmable using arrays of microdisk resonators.

## Introduction

With the exponential growth of digital data generated from high definition images, videos, and speech from sources, such as social media and the internet-of-things, there is an intense need for data analytics to make the data understandable and actionable. To serve these needs, big data analytics becomes an important and fast-growing emerging field, in which field-programmable gate arrays (FPGAs) are widely used for data processing, thanks to its uniqueness in fast reconfigurability^1,2^. However, the computational speed and power efficiency of the state-of-the-art FPGAs are limited due to the low electronic clock rates and high Ohmic losses. To overcome the limitations, photonics has been envisioned as an enabling technology^3,4^. In particular, photonic signal processing features ultra-wide bandwidth and ultra-fast computational speed with a low power consumption^5,6^. In the past 20 years, considerable progress has been made in the implementation of various photonic-assisted signal processing functions^7,8^. However, most of the demonstrated photonic solutions are realized with the use of discrete fiber-based components or devices, which make the system bulky and costly.

With the rapid development of photonic-integrated circuits (PICs), leveraging PICs technology to realize on-chip integration of photonic signal processors has been an active research field^9–12^. Recently, great effort has been directed to the study of PICs for microwave signal generation and processing^13,14^, and various solutions have been proposed and demonstrated based on different material platforms^15–19^. However, most of the reported approaches were implemented with task-oriented designs. For practical applications, a universal signal processor that can be re-programmed on demand, similar to an electronic FPGA-based signal processor, is highly desired. To meet this goal, extensive effort has been made to the implementation of photonic FPGA processors. Zhuang et al. proposed a photonic on-chip programmable signal processor that consists of a grid of tunable Mach–Zehnder interference (MZI) couplers interconnected in a two-dimensional mesh network^20^. Such a device is able to be programmed to present many different circuit topologies and thereby provides a diversity of signal processing functions. Inspired by this approach, photonic signal processors with other mesh topologies were also proposed, such as processors with hexagonal and triangular-shaped meshes^21,22^. Compared with an MZI coupler, a micro-ring or micro-disk resonator (MDR) has a much smaller footprint. Thus, the use of micro-ring or MDRs as a fundamental execution unit will make a signal processor have a much smaller size and stronger wavelength selectivity.^23^

In this paper, we report a scalable photonic integrated field-programmable disk array (FPDA) signal processor using MDRs as fundamental execution units for ultra-fast and programmable signal processing. With a fabricated 8 × 8 FPDA, photonic microwave signal-processing functions including reconfigurable wavelength multiplexing/demultiplexing, frequency-tunable flat-top filtering, tunable microwave delay, multichannel temporal differentiation, optical beamforming, and reconfigurable optical pulse shaping, are experimentally demonstrated.

## Results

### Chip design and measurement

**Fig. 1:**
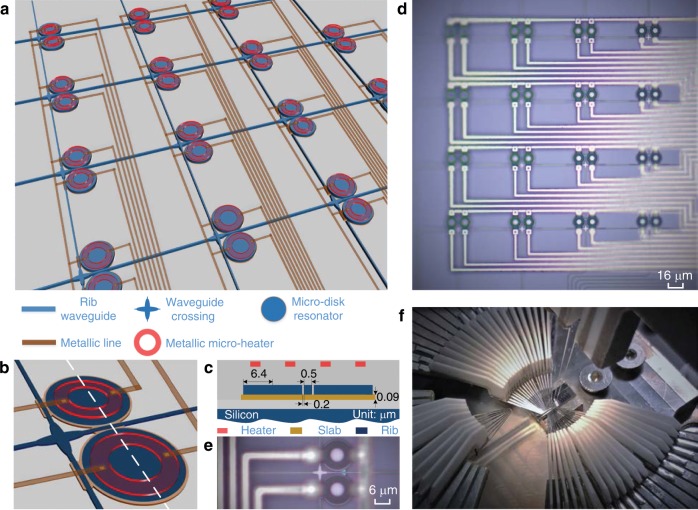
Scalable photonic integrated FPDA signal processor. **a** Schematic layout of the proposed FPDA signal processor. **b** Zoom-in view of a singe mesh cell. **c** Cross-sectional view of the planar structure of two disks along the white dash line in **b**. **d** Chip prototype of an 8 × 8 FPDA signal processor. **e** Zoom-in view of a single mesh cell. **f** Image of the chip characterization set-up.

The key components in the FPDA are the ultra-compact MDRs, which are used as the fundamental execution units in the core to route, store, and process optical signals. Figure 1a shows the proposed FPDA signal processor architecture on a silicon photonic chip, which has a two-dimensional mesh network structure with multiple input and multiple output ports. In each mesh cell, two identical thermally tunable high-Q MDRs are used to perform optical routing, storage, and processing, as shown in Fig. 1b, c illustrates the cross-sectional view of the planar structure of two disks along the white dash line in Fig. 1b, d is an image of a chip prototype of an 8 × 8 FPDA signal processor captured by a microscope camera (see “Methods” section for more details about the processor design and layout), and Fig. 1e gives a zoom-in view of single mesh cell, in which a low-loss and low-crosstalk waveguide crossing with a 1 × 1 multimode-interference (MMI) configuration is used for guiding the optical signal at the waveguide intersection^24^. Figure 1f is an image to show the chip characterization set-up. Two single-mode fibers are used for optical I/O coupling, and multiple DC probes are employed to apply voltages to the MDRs. By programming the voltages, the processor could be reconfigured to have diverse circuit topologies to perform multiple tasks, and each independent processing task is assigned to a dedicated section of the chip, which enables a strong parallel computing capacity.

Thanks to the high index contrast and compatibility with current CMOS technology, silicon is chosen as the material system to fabricate the proposed processor, which offers key advantages including small footprint and seamless integration with electronics. Optical performance evaluation of a single mesh cell is firstly performed in terms of optical routing and storage. The measurement results (see Supplementary Note 1) show that a single mesh cell can route an input optical signal to any desired output port or split an input optical signal into three at arbitrary splitting ratios, and a time delay up to 23 ps is generated by one MDR in the mesh cell. With a scalable two-dimensional mesh network, the proposed FPDA signal processor could be reconfigured to perform multiple signal-processing functions.

At the waveguide crossing, two MDRs are employed. By controlling the voltages to tune the resonance wavelengths of the two MDRs, all-directional optical routing, storage, and processing are implemented, which enable the processor to support feedforward and feedbackward operations. By programming the voltages, the photonic FPDA processor could be reconfigured to have diverse circuit topologies to perform multiple tasks. Here, six photonic signal-processing functions including reconfigurable wavelength multiplexing/demultiplexing, flat-top filtering, tunable microwave delay, multichannel temporal differentiation, beamforming, and programmable optical pulse shaping, are experimentally demonstrated. Note that each independent processing task could be assigned to a dedicated section of the chip, to enable a strong parallel computing capacity.

### Wavelength multiplexing and demultiplexing

**Fig. 2:**
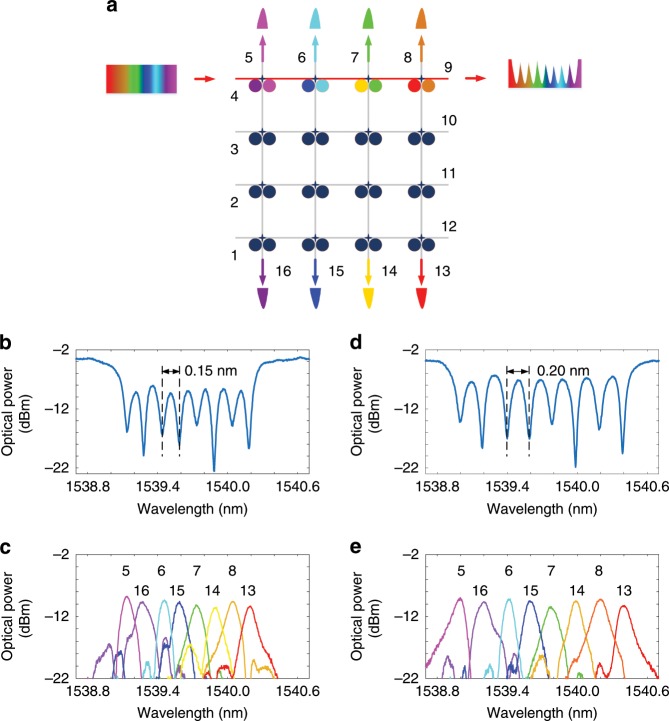
The FPDA reconfigured as a wavelength multiplexer/demultiplexer. **a** Schematic of the FPDA when operating as a wavelength demultiplexer. **b** Measured transmission spectrum at port 9 of the 8-channel demultiplexer with a channel spacing of 0.15 nm. **c** Measured transmission spectrum at the 8 drop ports (ports 5–8 and 13–16) of the demultiplexer with a channel spacing of 0.15 nm. **d** Measured transmission spectrum at port 9 of the 8-channel demultiplexer with a channel spacing of 0.2 nm. **e** Measured transmission spectrum at the 8 drop ports (ports 5–8 and 13–16) of the demultiplexer with a channel spacing of 0.2 nm.

As a key technology for expanding the capacity for optical communications networks, wavelength-division multiplexing (WDM) has been widely used in the present optical communications networks^25^. In a WDM system, a wavelength multiplexer/demultiplexer is a key optical component to multiplex or demultiplex different wavelengths corresponding to different channels. Thanks to the high wavelength selectivity and independent wavelength tuning of an MDR, the proposed FPDA processor could be used to operate as a wavelength multiplexer/demultiplexer with a tunable channel spacing at a low power consumption. Wavelength tuning of a single mesh cell in the proposed signal processor and its performance are evaluated (see Supplementary Note 1). Figure 2a shows the schematic of the processor when it is reconfigured to operate as a wavelength demultiplexer. An input optical signal is applied to the chip via port 4. By controlling each voltage to tune the resonance wavelength of each MDR along the channel from port 4 to port 9, the optical signal could be selected by a specified MDR and routed to a specified port. Figure 2b shows the measured transmission spectrum at port 9, in which an 8-channle demultiplexer with a uniform channel spacing of 0.15 nm is presented. All the notch depths are larger than 10 dB. The extinction ratio between the light at the drop channel and the remaining light in the pass port is 7 dB, which can be improved by reducing the insertion loss at the drop port. Figure 2c shows the transmission spectra at the eight drop ports, ports 5–8 and 13–16. The total power consumption is measured to be 29.4 mW. By tuning the voltages, the channel spacing could be tuned. Figure 2d shows the measured transmission spectrum at port 9 with a channel spacing of 0.2 nm, and Fig. 2e shows the measured transmission spectra at all the eight drop ports, again. The power consumption is measured to be 31.9 mW. Due to the fabrication imperfections, the selectivity of the MDRs is not uniform, which is caused by random sidewall roughness of the MDRs in the fabrication and could be alleviated using more advanced fabrication process with a higher precision and resolution. With the voltages further tuned, the channel spacing could be tuned to fit the standard WDM channel spacing grid. By programming all the MDRs, the FPDA processor could be reconfigured to be a versatile wavelength multiplexer/demultiplexer bank, with each channel being configured to operate for a different wavelength range, which is useful for future agile photonic networks.

### Flat-top filtering

**Fig. 3:**
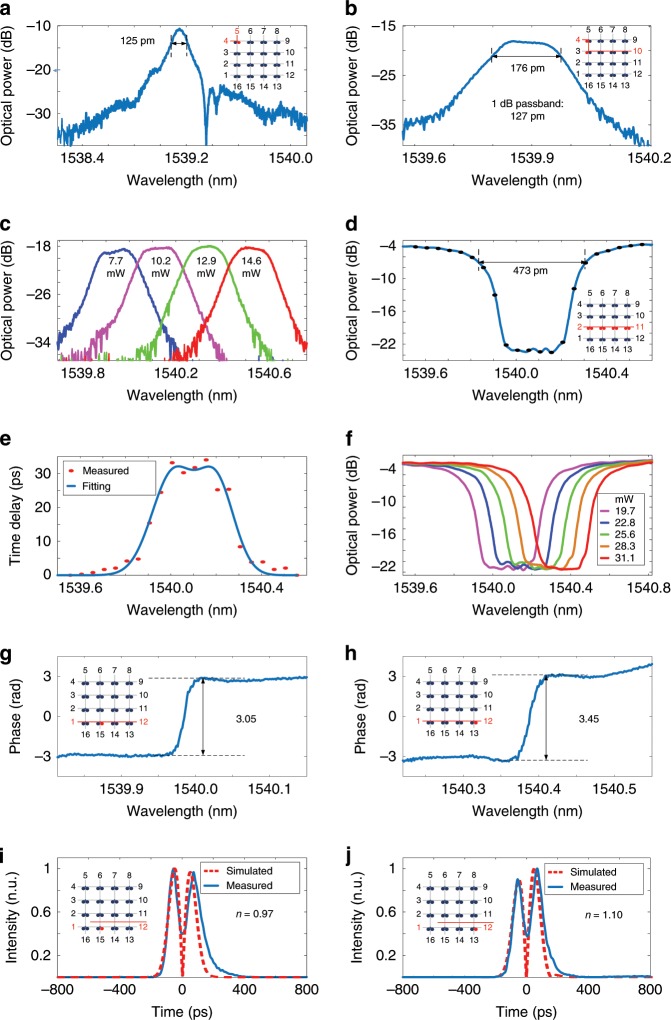
Experimental results with FPDA reconfigured to perform multiple functions. **a** Measured transmission spectrum at port 5 when an optical signal is applied to the chip via port 4. **b** Measured transmission spectrum at port 10 when the FPDA operates as a flat-top optical filter. **c** Center-frequency tuning of the flat-top optical filter. **d** Measured transmission spectrum at port 11 when an optical signal is applied to the chip via port 2. **e** Measured group delay. **f** Center-frequency tuning of the delay line. **g** Measured phase response of the 3rd MDR from the left in the channel from port 1 to port 12. **h** Measured phase response of the 8th MDR. **i** Measured differentiated Gaussian pulse when the 3rd MDR works as a temporal differentiator. **j** Measured differentiated Gaussian pulse when the 8th MDR works as a temporal differentiator.

Optical filters with a flat top can find wide applications in optical communications and interconnect systems for signal processing and routing^26,27^. The FPDA can be reconfigured as a flat-top optical filter. Figure 3a shows the measured transmission spectrum at port 5 when the input optical signal is applied to the processor via port 4. As can be seen, the optical filter has a 3-dB bandwidth of 125 pm and an out-of-band rejection ratio of 9 dB. The top of the optical filter can be made flat if two MDRs are cascaded with their resonance wavelengths slightly apart. In the experiment, the DC voltages are controlled to make the resonance wavelengths of the two MDRs have an offset wavelength of 0.08 nm, an optical filter with a flat top is produced. Figure 3b shows the measured transmission spectrum at port 10. As can be seen, an optical filter with a flat top is achieved. The 3-dB bandwidth is 176 pm. Considering that the 1-dB bandwidth is 127 pm, the out-of-band rejection ratio is calculated to be 17 dB, which is 8 dB higher than the optical filter using a single MDR. In addition to the control of the top flatness, the center wavelength of the optical filter can be tuned, which can be done by simultaneously tuning the resonance wavelengths of the two MDRs. Figure 3c shows the measured center-frequency tuning of the flat-top optical filter. As can be seen the center wavelength of the filter is red shifted by 590 pm. The power consumption due to the use of the micro-heaters is 14.6 mW. By incorporating more MDRs, a flat-top optical filter with a wider bandwidth and higher out-of-band rejection ratio can be achieved.

### Tunable microwave delay

Due to a strong light-confinement capacity of an MDR, a strong dispersion near the center of the notch is observed, corresponding to a large group delay. Thus, an MDR can be used as a compact optical delay line^28–30^. However, a single MDR usually has a group delay with a narrow bandwidth^28^. To increase the bandwidth, more MDRs can be cascaded with their center wavelengths slightly separated. For the fabricated FPDA, a tunable optical delay line using eight cascaded MDRs is implemented. Figure 3d shows the measured transmission spectrum at port 11 when an input optical signal is sent to the processor via port 2. The 3-dB bandwidth is measured to be 473 pm. The bandwidth of the delay line could be tailored on demand by increasing or decreasing the number of the MDRs. Figure 3e shows the measured group delay, in which a maximum time delay of 32 ps is achieved (see “Methods” section for more details about the time delay measurement). By increasing the radius of the disk, the optical-confining capacity of the MDR could be further enhanced, which is of help to increasing the time delay. Figure 3f shows the center-frequency tuning of the delay line by controlling the DC voltages to the MDRs. As can be seen, a red-shift in wavelength of 280 pm is observed. The applied electrical power to the micro-heaters is measured to be 31.1 mW, and the wavelength shift rate is calculated to be 24.6 pm/mW. If a FPDA with more mesh cells is used, optical delay lines with larger time delays and wider bandwidths can be implemented.

### Multichannel temporal differentiation

An all-optical multichannel temporal differentiator is a device that is capable of performing temporal differentiation of multichannel signals carried by multiple wavelengths and can be employed in a WDM system for ultrafast multichannel signal processing and characterization^31,32^. Thanks to the independent tuning of each MDR, a multichannel optical temporal differentiator can be implemented using the proposed FPDA. Figure 3g shows measured phase response of the third MDR from the left in the channel from port 1 to port 12. The phase jump is measured to be 3.05. Figure 3h shows the measured phase response of the eighth MDR in the same channel, and its phase jump is 3.45. When an optical Gaussian pulse with a full-width at half-maximum of about 85 ps falls into the resonance notch of an MDR, at port 12 a differentiated Gaussian pulse is generated after photodetection. Figure 3i shows a measured temporally differentiated pulse when the optical Gaussian pulse falls into the resonance notch of the third MDR, which works as a temporal differentiator with a differentiation order of 0.97 (blue-solid line). A simulated differentiated Gaussian pulse (red-dashed line) is also shown in Fig. 3i for comparison. The experimentally differentiated pulse is close to the simulated one, which confirms the effectiveness of the use of the device to perform fractional-order differentiation. Figure 3j shows the measured temporally differentiated pulse when the optical Gaussian pulse falls into the resonance notch of the eighth MDR, which works as a temporal differentiator with a differentiation order of 1.10 (blue-solid line). The differentiation orders are different for the two MDRs, which is caused by the disk non-uniformity induced by fabrication imperfections. By thermal tuning, the multi-channel temporal differentiator could be frequency-tunable and the channel-spacing can be reconfigurable, which is a promising device for future agile photonic network.

### Optical true time delay beamforming

**Fig. 4:**
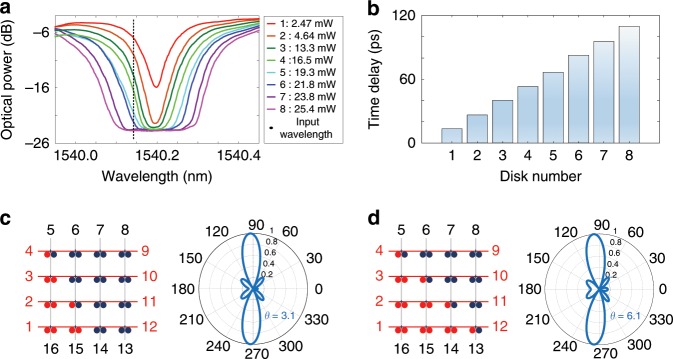
Experimental results with photonic FPDA signal processor operating as optical beamforming network. **a** Measured transmission spectrum of the channel from port 4 to port 9 when the voltages are controlled to make resonance wavelength of each MDR aligned progressively. **b** Measured time delays with the number of the aligned MDRs increasing progressively. **c** Calculated array factors of a four-element linear PAA when the channel time delay is 13.5 ps. **d** Calculated array factors of a four-element linear PAA when the channel time delay is 26.4 ps.

**Fig. 5:**
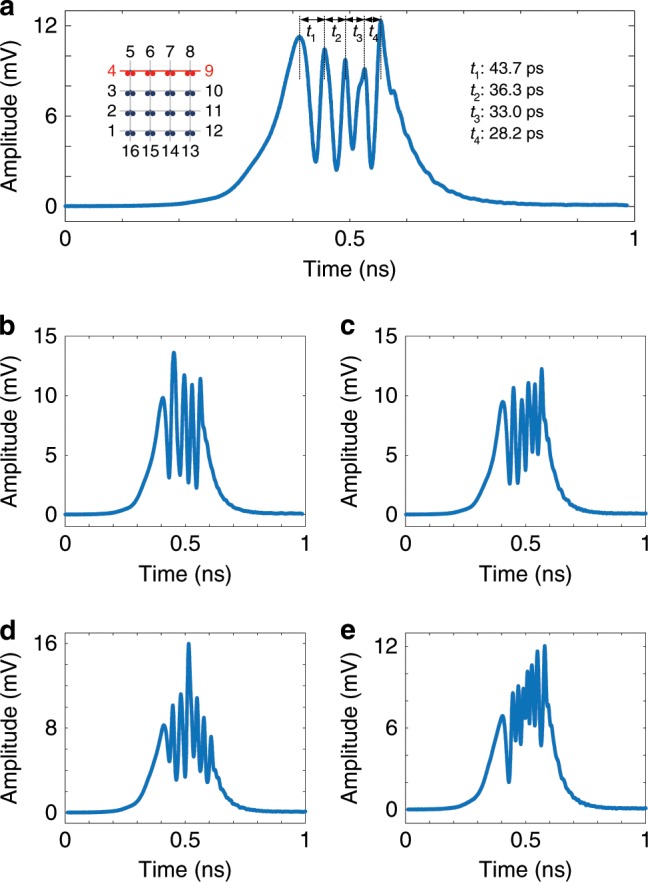
Experimental results with photonic FPDA signal processor operating as optical pulse shaper. **a** Generated microwave waveform with an up-chirped profile. **b**–**e** Generated microwave waveforms with reconfigurable peak number and amplitude.

An optical beamforming network is an attractive alternative to RF electronics in a phased array antenna (PAA), thanks to the large instantaneous bandwidth, small size, and low loss offered by photonics^33,34^. In the proposed FPDA, if a microwave signal carried by an optical carrier is applied to the chip via port 4, at the output port 9 progressive time delays can be generated by controlling the voltages to make resonance wavelengths of the MDRs aligned progressively. Thus, the FPDA can be used to implement a true-time delay beamforming network. Figure 4a shows the measured transmission spectrum of the channel from port 4 to port 9 when the voltages are tuned to make the resonance wavelengths of the MDRs aligned progressively. As observed, with the number of the aligned MDRs increasing, the notch depth is increased, and the bandwidth is also widened. In the meanwhile, it could be observed that the transmitted optical power is largely attenuated with more MDRs aligned. One solution is to heterogeneously integrate a III–V optical amplifier at the output of each MDR in the chip, to compensate for the optical loss. Figure 4b shows the measured time delays with the number of the aligned MDRs increasing progressively. The generated time delay is increased with the number of the aligned MDRs increasing. Especially, when the wavelengths of eight MDRs are aligned together, the total time delay is measured to be as large as 110 ps. Due to the progressive true-time delays offered by the MDRs, the FPDA can be reconfigured to operate as an optical beamforming network for PPA. Figure 4c, d shows the calculated array factors of a four-element linear PAA based on the fabricated signal processor as an optical beamforming network. The PAA has a uniform element spacing of half-wave length at a microwave frequency 2 GHz, and is driven by the time-delayed signals with different number of aligned MDRs in each channel. When the time delay difference between two adjacent channels is zero, the beam is pointing to the broadside direction; when the time delay difference between two adjacent channels is 13.5 ps, the beam pointing direction is 3.1^o^, as shown in Fig. 5c; when the time delay difference between two adjacent channels is increased to 26.4 ps, the beam pointing direction is 6.1^o^. Limited by the MDR number in each channel, in this demonstration the FPDA could provide a beam steering range from –6.1° to 6.1°. Thanks to the strong scalability of the FPDA, the number of the MDRs in each channel could be highly increased, which would lead to an increased steering range. For example, if the FPDA is scaled up to have 50 × 50 MDRs, a time delay difference as high as 220 ps between two adjacent channels is achieved, and thus a steering range from −60^o^ to 60^o^ would be produced.

### Arbitrary waveform generation

Spectral-shaping and wavelength-to-time (SS-WTT) mapping has been considered one of the most important photonic approaches to generate arbitrary microwave waveforms^35,36^. In a typical SS-WTT-mapping system, the spectral shaper is a key device which is specifically designed to have a spectral response with a shape that is a scaled version of the microwave waveform to be generated. Since the resonance wavelength of each MDR can be tuned independently, the FPDA can be reconfigured to operate as an optical pulse shaper. By independently controlling the DC voltages, an optical pulse shaper could be electrically reconfigured for arbitrary microwave waveform generation. In the demonstration, an ultra-short optical pulse is sent to the chip via port 4, and at the output port 9 a spectrally shaped optical pulse is generated, which is then sent to a 10 km single-mode fiber with a chromatic dispersion of 105.4 ps/nm to perform wavelength-to-time mapping. After photodetection, an arbitrary microwave waveform is generated. Figure 5a shows a generated microwave waveform that is linearly chirped, which is done by making the notch spacing between the MDRs linearly decreasing. Figure 5b–e shows the generated microwave waveforms by controlling the individual peak number and amplitudes within the waveform. Specifically, Fig. 5b shows the generated microwave waveform with a square profile of five peaks; Fig. 5c shows the generated microwave waveform with a square profile of six peaks; Fig. 5d shows the generated microwave waveform with a triangular profile; and Fig. 5e shows the generated microwave waveform with a sawtooth profile. The demonstration shows the capability of the processor to generate complex microwave waveform, which is beyond the capability of an electronic arbitrary microwave waveform generator and also shows the fast reconfigurability of the pulse shaper with the use of the FPDA. Compared with the previously reported on-chip optical pulse shaper working in the drop port^37,38^, our FPDA-based optical pulse shaper works at the transmission port, which holds key advantages in terms of low insertion loss and simple device configuration.

## Discussion

**Fig. 6:**
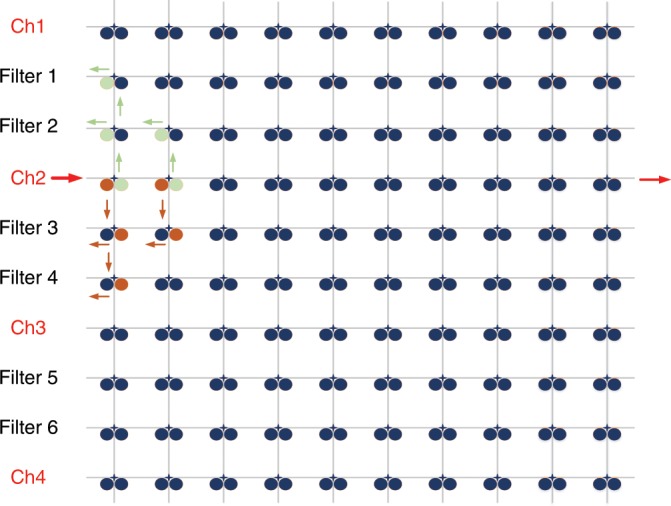
A 10 × 10 signal processor layout. When this processor is operating as an optical beamforming network, four channels are used as beam steering channels and six channels as filter channels to eliminate crosstalk.

Note that in Fig. 4, the light coming from port 4 can propagate to port 10 and will cause interference. For a large-scale processor, the crosstalk in the processor could be minimized by using filters to remove the crosstalk. Take a 10 × 10 signal processor as an example. When the processor is used to implement a 4-channel TTD beamforming network, one from every three channels can be selected to serve as a beam steering channel, and the other two channels are used as filters to filter out the leaked optical signal. As can be seen in Fig. 6, there are totally 10 channels including four beam steering channels and six filter channels. For example, when an optical signal is applied to Channel 2, at the waveguide crossing, due to two microdisk resonators some optical power will transmit to the neighboring channels. By using the MDRs as optical filters in the filter channels, the leaked optical signals could be filtered out. With this approach, the crosstalk will be significantly reduced.

To achieve large-scale integration, the insertion loss of the chip, especially the loss at a drop port, should be reduced. In addition, the spacing between two MDRs at a waveguide crossing needs to be increased by placing the MDRs at different coupling positions along the bus waveguide to further minimize the thermal crosstalk. Furthermore, by incorporating a tunable directional coupler in the MDR, the resonant wavelength, Q-factor, and extinction ratio could be tuned, which would offer more room to improve the chip performance. Table 1 shows the comparison between the reported works^20,21,39^ and our work. As can be seen, thanks to the compact size of the MDRs and the use of silicon photonics technology, the entire device has a footprint as small as 0.16 mm^2^, which enables the processor to have a strong scalability and low power consumption. In addition, due to the high wavelength selectivity of the MDRs, the chip could be reconfigured to be an optical pulse shaper used in photonic arbitrary waveform generation, which is a distinctive functionality. In future work, a largely scaled mesh structure including a tunable directional coupler in each MDR will be employed in the processor to enrich functionality and enhance performance.

**Table 1:** Comparison of reported programmable optical signal processor.

Year	Ref.	Material	Mesh structure	Size	Operation approach	Functionality
2015	^20^	Si_3_N_4_	Square	7 × 3.5 mm^2^	MZI coupler	-Notch filter -Hilbert transformer, -Bandpass filter, -Delay line
2016	^39^	III–V	Ring	1.5 × 2 mm^2^	Ring resonator	-Integrator -Differentiator -Hilbert transformer
2017	^21^	SOI	Hexagonal	15 × 20 mm^2^	MZI coupler	21 functionalities^a^
2019	Our work	SOI	Square	0.4 × 0.4 mm^2^	MDR	-Demultiplexer -Flat-top filter -Tunable delay line -Differentiator -Beamforming network -Optical pulse shaper

^a^21 different functionalities include unbalanced FIR Mach–Zehnder filters, ring cavities, complex CROW, SCISSOR, and ring-loaded MZI filters, and multiple input–multiple output linear-optic transformation devices, including a C-NOT gate

In conclusion, a silicon photonic FPDA signal processor was designed, fabricated, and characterized, and the use of the FPDA for advanced microwave signal generation and processing has been demonstrated. The processor has a two-dimensional mesh network structure, and in each mesh cell two identical thermally tunable high-Q MDRs were leveraged for optical routing, storage, and processing. By programming the MDRs, the FPDA could be reconfigured to have diverse circuit topologies to perform multiple signal-processing functions including filtering, temporal differentiation, time delay, beamforming, and spectral shaping. Each independent processing function could be done using a dedicated section of the chip, which would lead to a strong parallel computing capacity of the signal processor. Thanks to the compact size and strong wavelength selectivity of the MDRs, the device has the key advantages including high scalability, instant and flexible reconfigurability, strong parallel computing capability, and low power consumption.

With a fabricated 8 × 8 FPDA, a few photonic microwave signal-processing functions were experimentally demonstrated including reconfigurable wavelength multiplexing/demultiplexing, frequency-tunable flat-top filtering, tunable microwave delay, multichannel temporal differentiation, optical beamforming, and reconfigurable optical pulse shaping. Thanks to the simple mesh structure architecture, the processor could be made to have a large scale, which would create more functionalities with better performance for programmable photonic signal processing.

## Methods

### FPDA design and layout

The silicon photonic-integrated FPDA signal processor has a two-dimensional mesh network structure with eight input and eight output ports. The mesh cell has a square shape having a length of 75 μm. In each mesh cell, two identical thermally tunable high-Q MDRs are leveraged to route, store and process optical signals, and a low-loss waveguide crossing based on a 1 × 1 MMI is employed at the waveguide intersection to enable low-crosstalk horizontal and vertical optical transmission. To reduce the sidewall roughness, in order to increase the light confinement capacity and optical coupling between the bus waveguide and the disk, each MDR in the FPDA is designed to have an additional slab waveguide to wrap the disk and bus waveguide. In the mesh cell, as shown in Fig. 1b, c, two identical MDRs are employed to operate in an add-drop fashion. The parameters of the MDRs in the mesh: the disk radius is 6.4 µm and the disk height is 220 nm. The height of the bus waveguides is also selected to be 220 nm and the height of the additional slab waveguide is selected to be 90 nm. Note that the widths of the slab waveguide wrapping the disk and the bus waveguide are controlled to 200 nm. To enable effective coupling, the two slab waveguides in the coupling region are designed such that they fully overlap. The bus waveguide has a wider width of 500 nm, to ensure an effective excitation of the first-order WGM. To enable thermal tuning, each MDR has a high-resistivity metallic micro-heater, placed on top of the disk. The MMI-based crossing consists of two orthogonal intersecting MMI waveguides with each having a width of 1.44 μm and a length of 6 μm, to enable low-loss and low-crosstalk transmission. The chip design was sent to the AMF in Singapore for fabrication with the 248-nm lithography process.

### Temperature-stabilized setup

A thermoelectric-cooler (TEC) on which the silicon chip was located was used in order to control and stabilize the chip temperature. Adjacent to the silicon chip, a thermistor was placed to measure and provide a feedback temperature to a commercial TEC controller which was used to control and stabilize the chip temperature at 23 °C during the experiment.

### Time delay measurement

A microwave rectangular pulse with a temporal width of 3 ns was generated using an arbitrary waveform generator (Keysight, M8195A) and sent to an intensity modulator (Optilab, IM-1550-40-PM), where an input optical signal from a laser source (Keysight, N7714A) was modulated by the microwave rectangular pulse. Then, the modulated optical signal was applied to chip, and at the output the optical signal was guided to a photodetector (Optilab, LR-12-A-M), where the microwave rectangular pulse was recovered. A real-time oscilloscope (Keysight, Infiniium Z-Series 160 GSa/s) was employed to capture the temporal waveform of the recovered microwave pulse. By locating the optical signal wavelength at the different positions around the resonance notch of the MDR, different time delay of the microwave pulse could be observed with the oscilloscope.

## Supplementary information

Supplementary Information

## Data Availability

The data that support the findings of this study are available from the corresponding author upon request.
