# Supplementary material for: Photonic integrated field-programmable disk array signal processor
Source: Nat Commun. 2020 Jan 21;11:406. doi: 10.1038/s41467-019-14249-0 (PMC6972927; doi:10.1038/s41467-019-14249-0)
Supplement: Supplementary file 1 — Supplementary Information [file 41467_2019_14249_MOESM1_ESM.pdf]

SUPPLEMENTARY INFORMATION

Photonic integrated field-programmable disk array signal processor

Zhang et al.

Photonic integrated field-programmable disk array signal processor

Weifeng Zhang and Jianping Yao*

Microwave Photonic Research Laboratory, School of Electrical Engineering and Computer Science, University of Ottawa, 25 Templeton Street, Ottawa, Ontario, Canada K1N 6N5

Supplementary Note 1: single mesh cell performance evaluation

Optical performance evaluation of a single mesh cell is performed in terms of optical routing and storage. Supplementary Figure 1a shows the schematic of a single mesh cell. Supplementary Figure 1b shows a microscope image of the fabricated single mesh cell. Four grating couplers with a center-to-center spacing of 127 μm for coupling light into or out of the chip are used. Supplementary Figure 1c shows a zoom-in view of the waveguide intersection. A low-loss waveguide crossing based on 1×1 MMI is employed at the waveguide intersection to enable low-crosstalk optical transmission, and two tunable micro-disk resonators (MDRs) are employed to enable optical routing and storage.

When an input optical signal is launched from port 1 to the chip, owing to the high wavelength selectivity of the MDRs, the input optical signal would be routed to any desired output port or split into three signals at three output ports at arbitrary splitting ratios by independently controlling the DC voltages applied to the MDRs. The transmission spectra are measured using an optical vector analyzer (LUNA OVA CTe). Supplementary Figure 1d-f shows the measured transmission spectra at the three output ports. Supplementary Figure 1d shows the transmission spectrum at the output port 2, Supplementary Figure 1e shows the transmission spectrum at the output port 3, and Supplementary Figure 1f shows the transmission spectrum at the output port 4. As can be seen, at output port 3, transmission notches are observed, while at the other two output ports, port 2 and 4, transmission peaks occur. The insertion losses at the three ports are different since the lightwave signal travel through different paths. At the port 3, the insertion loss is measured to be -0.8 dB, which excludes which consists of the fiber-to-fiber I/O coupling loss of 14.6 dB, while at the port 2 and 4, the insertion loss is as large as -9 dB. In the transmission spectra, Each notch or peak in the spectrum represents a specific $WGM_{p,q}$ resonance in which *p* and *q* are the radial and azimuthal harmonic numbers. The periodicity of the notches and peaks tells that first-order and second-order whispering gallery-modes (WGMs) are effectively excited in the disks. The first-order WGM is measured to have a free spectral range (FSR) of 17.5 nm, and the second-order WGM has an FSR of 18.3 nm. Then, when the input optical signal is launched from port 2 to the chip, the transmission spectra are measured at the output port 3 and 4. Since the optical transmission between the port 1 and port 2 is symmetric, the transmission spectrum at the port 1 is identical to that shown in Supplementary Figure 1e. Supplementary Figure 1g and 1h shows the transmission spectrum at the output port 3 and 4, respectively. In Supplementary Figure 1g, the peaks are caused by the selectivity of disk 2, and in Supplementary Figure 1h, the notches are resulted by the light-confining capacity of the two disks. When the input optical signal is launched from port 3 to the chip, the transmission spectrum is measured at the output port 4. In Supplementary Figure 1g, the peaks are caused by the selectivity of disk 1.

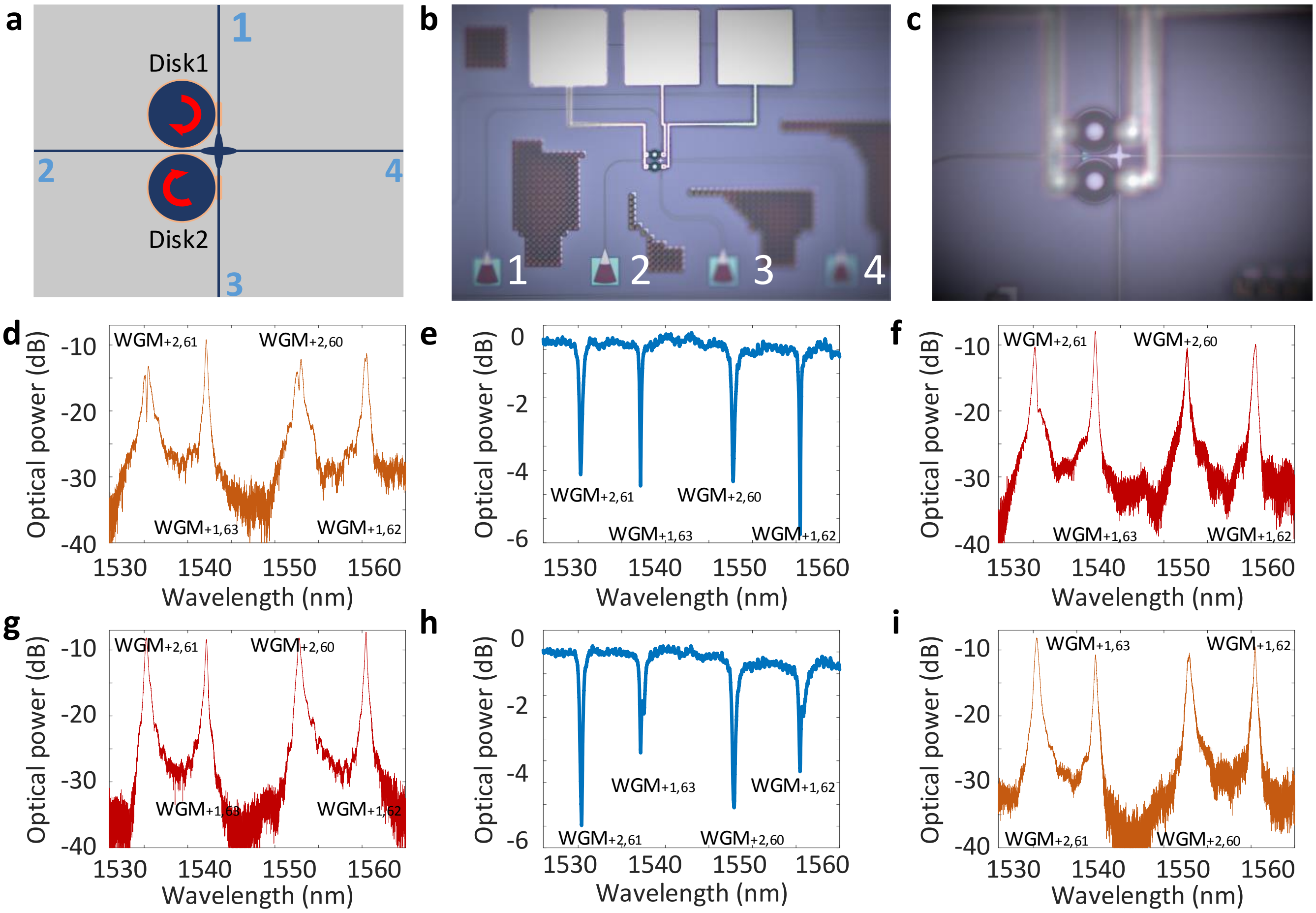

<u>Supplementary Figure 1</u> Performance evaluation of a single mesh cell in the photonic FPDA signal processor. a, Schematic of the single mesh cell. **b**, Chip prototype of the single mesh cell. **c**, Zoom-in view of the thermally-tunable MDRs and waveguide crossing. **d**, Measured transmission spectrum from port 1 to port 2. **e**, Measured transmission spectrum from port 1 to port 3. **f**, Measured transmission spectrum from port 1 to port 4. g, Measured transmission spectrum from port 2 to port 3. **h**, Measured transmission spectrum from port 2 to port 4. **i**, Measured transmission spectrum from port 3 to port 4.

By applying a DC voltage to the metallic micro-heater on top of each disk, the heat will be generated and based on thermal-optic effect, the refractive index of silicon will be increased, which leads to the resonance wavelength shifting of each MDR. Firstly, a DC voltage is applied to disk 1 and is increased. <u>Supplementary Figure 2a and 2b</u> shows the measured transmission spectral shift of the channel from port 3 to port 4, and port 3 to port 2, respectively. To clearly shows the wavelength shift, the two figures shows zoom-in view of the $WGM_{+2,61}$ resonance. <u>As can be seen</u>, as the DC power is increased, the resonance wavelengths of the two MDRs are red-shifted. From the voltage-current measurement, the micro-heater resistance is estimated to be 79.7 Ω. With the micro-heater power increasing from 0 to 6.10 mW, the resonance wavelength of the disk 1 is red-shifted by 1.26 nm, and the wavelength shift rate is calculated to be 0.21 nm/mW. Due to the thermal crosstalk, with the voltage increasing, the resonance wavelength of the disk 2 is red-shifted by 0.32 nm, and the wavelength shift rate is calculated to be 0.05 nm/mW, which is much less than that of the disk 1 thanks to the key advantages of the MDRs including compact footprint and strong light-confining capacity over the micro-ring resonators. However, the thermal crosstalk is absolutely unwanted. To minimize the crosstalk, one solution is to increase the spacing between the two disks by allocating the disks at the different coupling positions along the bus

waveguide. In addition, during the wavelength tuning, the insertion loss, extinction ratio and the Q-factor of the resonance remains unchanged. Then, the DC voltage is applied to disk 2 and is increased. Supplementary Figure 2c and 2d shows the measured transmission spectral shift of the channel from port 3 to port 4, and port 3 to port 2, respectively. Again, as the DC power is increased, the resonance wavelengths of the two MDRs are red-shifted. From the voltage-current measurement, the micro-heater resistance is estimated to be 65.6 Ω. The resistance difference between the two top-placed micro-heater is due to the fabrication imperfections. With the micro-heater power increasing from 0 to 7.39 mW, the resonance wavelength of the disk 2 is red-shifted by 1.90 nm, and the wavelength shift rate is calculated to be 0.26 nm/mW. Due to the thermal crosstalk, with the voltage increasing, the resonance wavelength of the disk 1 is red-shifted by 0.41 nm, and the wavelength shift rate is calculated to be 0.06 nm/mW, which is much less than that of the disk 2. Again, to minimize the crosstalk, one solution is to increase the spacing between the two disks. Thanks to the thermal tuning, the two MDRs could be tuned to realize the optical routing and during the tuning the insertion loss, extinction ratio and the Q-factor of the resonance remains unchanged.

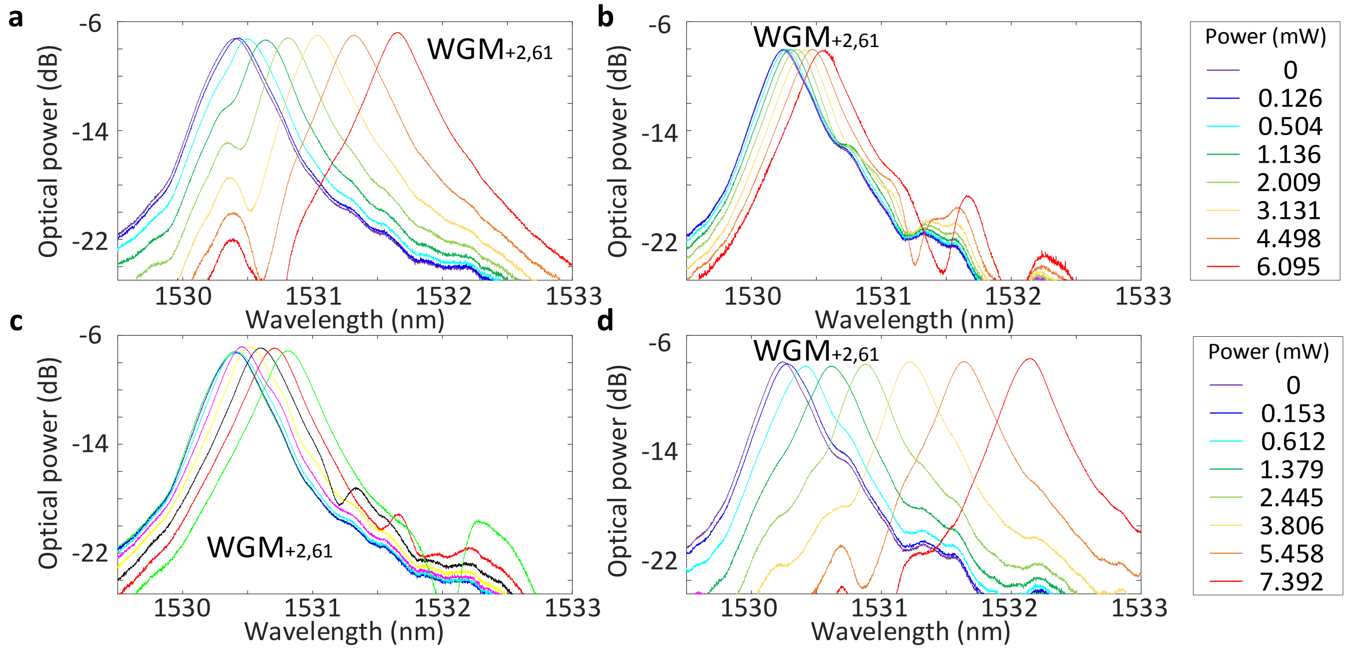

Supplementary Figure 2 Wavelength tuning of a single mesh cell in the photonic FPDA signal processor. a, Measured wavelength tuning of the resonance $WGM_{+2,61}$ from port 3 to port 4 when a DC power is applied to disk 1. **b**, Measured wavelength tuning of the resonance $WGM_{+2,61}$ from port 3 to port 2 when a DC power is applied to disk 1. **c**, Measured wavelength tuning of the resonance $WGM_{+2,61}$ from port 3 to port 4 when a DC power is applied to disk 3. **d**, Measured wavelength tuning of the resonance $WGM_{+2,61}$ from port 3 to port 2 when a DC power is applied to disk 2.

By applying two DC voltages to the two micro-heaters simultaneously, the input optical signal would be routed to any desired output port or split into three signals at three output ports at arbitrary splitting ratios by independently controlling the DC voltages applied to the MDRs. First, two DC voltages are controlled to make the two MDRs to operate at different resonance wavelengths. The spectra at output ports 2, 3 and 4 are measured when the total DC power consumption is 1.6 mW. The zoom-in views of the resonance $WGM_{+1,63}$ are shown in Supplementary Figure 3a-c, respectively. As can be seen, the disk 1 has a resonance wavelength at 1547.12 nm, while the disk 2 has a resonance wavelength at 1544.45 nm. Two MDRs has absolutely different resonance wavelengths, which could be used to route the input

optical signal to a desired output. Then, the DC voltages are changed to make the MDRs to operate at an identical wavelength. The transmission spectra at output port 2, 3 and 4 are again measured when the total DC power consumption is 2.4 mW. The zoom-in views of the resonance $WGM_{+1,63}$ are shown in Supplementary Figure 3d-f, respectively. As can be seen, the two MDRs have an identical resonance wavelength at 1543.65 nm, which split the input optical signal equally into two signals (ports 2 and 4). Thanks to the high selectivity and independent tunability of each MDR, a single mesh cell can route an input optical signal to any desired output port or split an input optical signal into three at arbitrary splitting ratios.

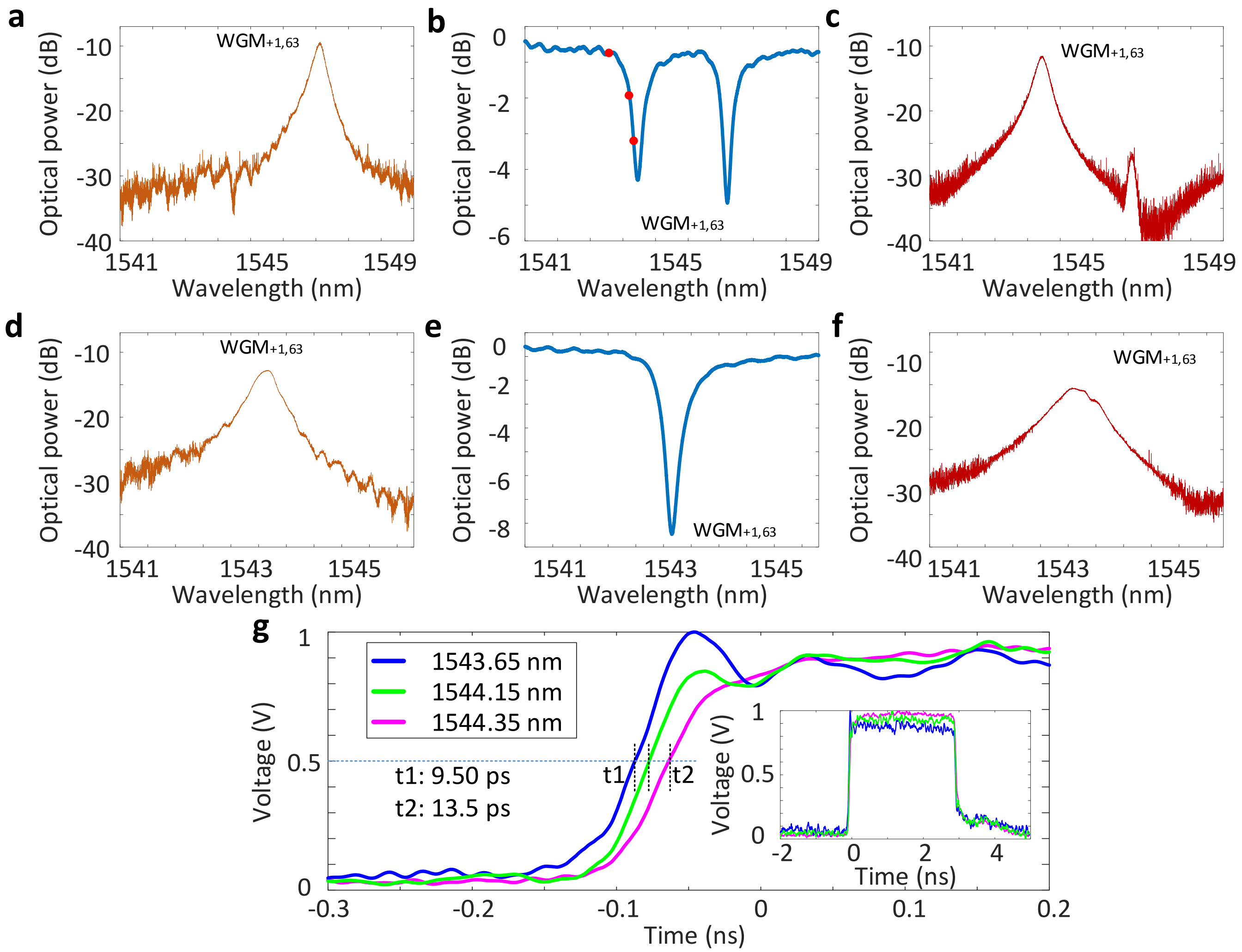

Supplementary Figure 3 Performance evaluation of a single mesh cell in the photonic FPDA signal processor. a-c, Zoom-in view of the resonance $WGM_{+1,63}$ when a total DC power consumption is 1.6 mW at the output port of 2, 3 and 4, respectively. **d-f**, Zoom-in view of the resonance $WGM_{+1,63}$ when a total DC power consumption is 2.4 mW at the output port of 2, 3 and 4, respectively. **g**, Measured time delay generated by one MDR.

In addition, due to the strong light-confinement capability of the optical cavity, the MDR has strong chromatic dispersion near the center of a notch and thus a large group delay, which is useful for optical storage. Supplementary Figure 3g shows the measured time delays at port 3 when the input optical signal with different wavelengths at 1543.65, 1544.15 and 1544.25 nm is applied to the chip via port 1. With the wavelength of the optical signal approaching the resonance wavelength of one MDR, the

measured time delay is increased, and a total time delay up to 23 ps is generated by one MDR. With more MDRs, the generated time delay could be further increased.

In summary, thanks to high wavelength selectivity and independent wavelength tunability, a mesh cell could be used to perform optical routing and storage. With a two-dimensional mesh network, the proposed FPDA signal processor could be reconfigured to perform multiple signal processing functions.
